# β1- and β3- voltage-gated sodium channel subunits modulate cell surface expression and glycosylation of Na_v_1.7 in HEK293 cells

**DOI:** 10.3389/fncel.2013.00137

**Published:** 2013-08-30

**Authors:** Cédric J. Laedermann, Ninda Syam, Marie Pertin, Isabelle Decosterd, Hugues Abriel

**Affiliations:** ^1^Pain Center, Department of Anesthesiology, University Hospital Center and University of LausanneLausanne, Switzerland; ^2^Department of Clinical Research, University of BernBern, Switzerland; ^3^Department of Fundamental Neurosciences, University of LausanneLausanne, Switzerland

**Keywords:** voltage-gated sodium channels (Na_v_s), Na_v_s β-subunits, glycosylation, biophysical properties, trafficking

## Abstract

Voltage-gated sodium channels (Na_v_s) are glycoproteins composed of a pore-forming α-subunit and associated β-subunits that regulate Na_v_ α-subunit plasma membrane density and biophysical properties. Glycosylation of the Na_v_ α-subunit also directly affects Na_v_s gating. β-subunits and glycosylation thus comodulate Na_v_ α-subunit gating. We hypothesized that β-subunits could directly influence α-subunit glycosylation. Whole-cell patch clamp of HEK293 cells revealed that both β1- and β3-subunits coexpression shifted *V*_½_ of steady-state activation and inactivation and increased Na_v_1.7-mediated *I*_Na_ density. Biotinylation of cell surface proteins, combined with the use of deglycosydases, confirmed that Na_v_1.7 α-subunits exist in multiple glycosylated states. The α-subunit intracellular fraction was found in a core-glycosylated state, migrating at ~250 kDa. At the plasma membrane, in addition to the core-glycosylated form, a fully glycosylated form of Na_v_1.7 (~280 kDa) was observed. This higher band shifted to an intermediate band (~260 kDa) when β1-subunits were coexpressed, suggesting that the β1-subunit promotes an alternative glycosylated form of Na_v_1.7. Furthermore, the β1-subunit increased the expression of this alternative glycosylated form and the β3-subunit increased the expression of the core-glycosylated form of Na_v_1.7. This study describes a novel role for β1- and β3-subunits in the modulation of Na_v_1.7 α-subunit glycosylation and cell surface expression.

## Introduction

Voltage-gated sodium channels (Na_v_s) are large glycoprotein complexes responsible for the initial rising phase of the action potential in excitable cells. They are composed of a highly processed α-subunit and are associated to one or more β-subunits (Brackenbury and Isom, 2011). The α-subunit is the pore-forming unit of the channel through which the Na^+^ ions pass (Catterall, 2000). Nine genes encoding Na_v_ α-subunits have been found in the human genome. In addition, four genes coding for the different Na_v_ β-subunits have been identified: *SCN1B* (Isom et al., 1992; Kazen-Gillespie et al., 2000), *SCN2B* (Isom et al., 1995a), *SCN3B* (Morgan et al., 2000) and *SCN4B* (Yu et al., 2003) coding for β1- to β4-subunits, respectively. The α-subunit is composed of four homologous domains (Noda et al., 1984). Each of these domains contains six α-helical transmembrane domains (S1–S6). S1–S4 form the voltage-sensing domains and thus regulate α-subunit opening. S5 and S6 form the pore of the channel (Guy and Seetharamulu, 1986; Payandeh et al., 2011). The pore-forming α-subunit permits the flow of Na^+^, but its biophysical properties are modulated by the β-subunits (Isom et al., 1995b), most likely via direct interference with gating (Zimmer and Benndorf, 2002). The influence of β-subunits on the biophysical properties of the recorded sodium current (*I*_Na_) vary with cell type, possibly due to different endogenous β-subunit expression and the presence of different partner proteins (Meadows and Isom, 2005). The β-subunits also participate in cell–cell adhesion and cell migration via the interaction with the extracellular matrix and cytoskeletal molecules. They also serve as important signaling molecules (Isom, 2001). Naturally occurring genetic variants in humans and genetically modified animal models have shown that β-subunits are implicated in numerous diseases, i.e., pain, epilepsy, migraines and cardiac arrhythmias (Brackenbury and Isom, 2011). This highlights their importance in the regulation of cellular excitability. The β-subunits are composed of an extracellular immunoglobulin-like domain in the N-terminal region, a single transmembrane segment and an intracellular carboxy-terminus tail (Isom et al., 1992). β1- and β3-subunits interact with the α-subunit via a non-covalent bond (Hartshorne et al., 1982), while β2- and β4-subunits are covalently linked to the α-subunit via disulfide bonds (Hartshorne et al., 1982; Messner and Catterall, 1985; Yu et al., 2003).

Out of the total pool of Na_v_s, most of the α-subunits are localized intracellularly: in the endoplasmic reticulum (ER) for synthesis, in the Golgi where post-translational modifications occur and in the secretory pathway where they are trafficked to the plasma membrane to exert their main functions (Schmidt et al., 1985; Ritchie et al., 1990; Okuse et al., 2002). In the ER and the Golgi, Na_v_ α-subunits undergo extensive sequential glycosylation (Waechter et al., 1983; Schmidt and Catterall, 1987), a process involving the addition of N-acetylglucosamine capped by sialic acid residues and the sequential addition of oligosaccharide chains. Glycosylation can account for up to 30% of the α-subunit molecular weight (Messner and Catterall, 1985). Protein glycosylation serves various functions such as protein folding, cell signaling, protection from proteases, cell-cell adhesion and regulation. It has also been implicated in development and immunity (Moremen et al., 2012). Glycosylation modifies the gating properties of the Na_v_ α-subunits (Recio-Pinto et al., 1990; Bennett et al., 1997; Zhang et al., 1999; Tyrrell et al., 2001), most likely by interfering with the electric field near the gating sensors (Bennett et al., 1997; Cronin et al., 2005; Ednie and Bennett, 2012).

Because both β-subunits and glycosylation modify the intrinsic biophysical properties of the Na_v_ α-subunit, we hypothesized that β-subunits might directly influence α-subunit glycosylation. This study investigated the effect of the four β-subunits on the Na_v_1.7-mediated current when co-expressed in HEK293 cells. Each of the four β-subunits influenced the biophysical properties and kinetics of the Na_v_1.7-mediated current to varying degrees, but only the β1- and β3-subunits increased Na_v_1.7 current density. Cell surface biotinylation and subsequent deglycosylation of the samples revealed the presence of differentially glycosylated forms of Na_v_1.7 in the cell; a core-glycosylated and a fully-glycosylated form of Na_v_1.7. β1- and β3-subunits mediated the differentially glycosylated form of Na_v_1.7 and enhanced its expression at the membrane. This suggests that the increase in Na_v_1.7 *I*_Na_ may be explained by a glycosylation-dependent stabilization of Na_v_1.7 at the cell membrane. This work reveals a novel mechanism by which Na_v_ β-subunits modulate α-subunit glycosylation and cell surface density.

## Materials and methods

### DNA constructs

Na_v_1.7, β1-, β2-, and β4-subunit cDNA cloned into pCINh and β3-subunit cloned into pFBM were provided by Dr. S. Tate (Convergence Pharmaceuticals, Cambridge, UK).

### Cell culture and transfection

Human embryonic kidney (HEK293) cells were cultured in DMEM medium supplemented with 10% FBS, 4 mM Glutamine and 20 μg/ml Gentamicin, at 37°C in a 5% CO2 incubator (Life Technologies Inc.). For patch clamp experiments, 1 μ g of Na_v_1.7 cDNA concomitantly with 0.4 μ g of a β-subunit and 0.8 μ g EBO-pCD-Leu2-CD8 cDNA encoding CD8 antigen as a reporter gene were transfected using the Ca^2+^-phosphate method in a T25 (~2 × 10^6^ cells). For biotinylation and deglycosylation assays, HEK293 cells were transiently co-transfected with 6 μ g of Na_v_1.7 and 6 μ g of each β-subunit or empty vector mixed with 30 μ l JetPEI (Polyplus-Transfection) and 250 μ l 150 mM NaCl in a P100 dish (~9 × 10^6^ cells, BD Falcon). For a negative control, the cells were transfected with 12 μ g of empty vector. The cells were used in patch clamp or biochemical experiments 48 h post transfection.

### Cell surface biotinylation assay

HEK293 cells transiently co-transfected were treated with 0.5 mg/ml EZ-link™ Sulfo-NHS-SS-Biotin (Thermo Scientific) in cold 1X PBS for 15 min at 4°C. The cells were then washed twice with 200 mM Glycine in cold 1X PBS to inactivate biotin, and twice with cold 1X PBS to remove excess biotin. The cells were then lysed with 1X lysis buffer [50 mM HEPES pH 7.4; 150 mM NaCl; 1.5 mM MgCl_2_; 1 mM EGTA pH 8; 10% Glycerol; 1% Triton X-100; 1X Complete Protease Inhibitor Cocktail (Roche)] for 1 h at 4°C. Whole cell lysates were centrifuged at 16,000 *g* at 4°C for 15 min. 2 mg of the supernatant was incubated with 50 μ l Streptavidin Sepharose High Performance beads (GE Healthcare) for 2 h at 4°C, and the remaining supernatant was kept as input. The beads were subsequently washed five times with 1X lysis buffer before elution with 50 μ l of 2X NuPAGE sample buffer (Invitrogen) and 100 mM DTT at 37°C for 30 min. These biotinylated fractions were analyzed as Na_v_1.7 expression at the cell surface. The input fractions, representing total expression of Na_v_1.7, were resuspended with 4X NuPAGE sample buffer plus 100 mM DTT to give a concentration of 1 mg/ml (60 μ g/well) and were then incubated at 37°C for 30 min.

### Deglycosylation assay

For the total fractions, 60 μ g of proteins of whole cell lysates were denatured at 37°C for 30 min in the presence of 1X Glycoprotein denaturing buffer. The denatured protein lysates were subsequently incubated at 37°C for 1 h with 1500 units PNGaseF (New England Biolabs) in the presence of 1X NP-40 and 1X G7 buffer to cleave most of the high mannose, hybrid and complex oligosaccharides from N-linked glycoproteins. The reaction was stopped by adding 4X NuPAGE sample buffer plus 100 mM DTT and incubating them at 37°C for 30 min. For the biotinylated fractions, 35 μ l ddH_2_O were added into Streptavidin Sepharose High Performance beads previously incubated with whole cell lysate and denatured at 37°C for 30 min in the presence of 1X glycoprotein denaturing buffer. The denatured proteins bound to Streptavidin beads were subsequently incubated at 37°C for 1 h with 2000 units of PNGaseF in the presence of 1X NP-40 and 1X G7 buffer. Following this incubation step, the beads were washed five times with the same lysis buffer used in the biotinylation assay and eluted with 2X NuPAGE sample buffer and 100 mM DTT at 37°C for 30 min.

### Western blots

Protein samples were separated on a 5–15% polyacrylamide gradient gel and blotted onto a nitrocellulose membrane using TransBlot Turbo transfer system (Biorad, Hercules). Antibody detections were performed in the SNAP i.d. system (Millipore) using the following antibodies: mouse monoclonal anti-Na_v_1.7 clone N68/6 (UC Davis/National Institute of Health (NIH) NeuroMab Facility, University of California), mouse monoclonal clone 464.6 anti-Na^+^/K^+^ ATPase α-1 (Abcam), rabbit polyclonal anti-actin A2066 (Sigma) and rabbit polyclonal anti-β4 (EnoGene). Rabbit polyclonal homemade anti-β1, anti-β2 and anti-β3 antibodies were provided by Dr. S. Tate (Convergence Pharmaceuticals, Cambridge, UK). Infrared IRDyeTM (680 or 800 CW)-linked goat anti-rabbit or anti-mouse IgG (LI-COR Biosciences) was used as secondary antibody. The blots were revealed and quantified with Odyssey Li-Cor (Lincoln).

### Electrophysiology

Twenty-four hours after transfection, cells were split at low density and whole-cell recordings were performed 48 h after transfection. Anti-CD8 beads (Dynal, Oslo, Norway) were used to identify transfected cells. Whole cell patch-clamp recordings were carried-out using an internal solution containing 60 mM CsCl, 70 mM Cs Aspartate, 11 mM EGTA, 1 mM MgCl_2_, 1 mM CaCl_2_, 10 mM HEPES, and 5 mM Na_2_-ATP, pH 7.2 with CsOH and an external solution containing 130 mM NaCl, 2 mM CaCl_2_, 1.2 mM MgCl_2_, 5 mM CsCl, 10 mM HEPES, 5 mM glucose, pH 7.4 with CsOH. Data were recorded with a VE-2 amplifier (Alembic Instruments, Montreal, Canada) or an Axon amplifier 700A and analyzed using pClamp software (version 8, Molecular Devices), Kaleidagraph (version 4.03) and MatLab. The sampling interval was set to 5 μ s (200 kHz) and low-pass filtering to 5.0 kHz. Resistance of the borosilicate pipettes (World Precision Instruments, Sarasota, FL, USA) was 2–6 MΩ. Leakage current was subtracted using the P/4 procedure. *I*_Na_ densities (pA/pF) were obtained by dividing the peak *I*_Na_ by the cell capacitance obtained from the pClamp function. Voltage dependence of activation (SSA) curves were determined from *I/V* curves where the Na^+^ current was evoked from a holding potential of −100 mV to test pulses of 100 ms ranging from −120 to +30 mV in increments of 5 mV. The linear ascending segment of the *I/V* relationship was used to estimate the reversal potential for each trace. Time constant of inactivation was determined by fitting the current decay with the Levenberg-Marquardt single exponential function. The time constant was plotted against the test voltage, with *I* = *A* * exp (−*t*/τ) + C: where *I* is the current, *A* is the percentage of channel inactivation with the time constant τ, *t* is time and C if the steady-state asymptote. Steady-state inactivation curves (SSI) were measured from a holding potential of −120 mV using 500 ms prepulses to the indicated potentials, followed by a test pulse to 0 mV. To quantify the voltage-dependence of SSA and SSI, data from individual cells were fitted with the Boltzmann relationship, *y* (*V_m_*) = 1/(1 + exp[(*V_m_*–*V*_½_)/*k*]), in which *y* is the normalized current or conductance, *V_m_* is the membrane potential, *V*_½_ is the voltage at which half of the available channels are inactivated and *k* is the slope factor.

Recovery from inactivation curves (RFI or “repriming”) were obtained with a standard two-pulse protocol consisting of a depolarizing pulse from a holding potential of −120 to 0 mV for 50 ms to inactivate the channels, followed by a variable duration (from 0.5 to 3000 ms) step back to −120 mV to promote recovery. Channel availability was assessed with the first standard test pulse at 0 mV. The normalized currents of the second pulse at 0 mV were plotted against the recovery interval. We calculated *t*_½_ (ms), the time necessary for half of the channels to recover from the first pulse, by interpolation from a linear relation between the 2 points juxtaposing half recovery (*y*_1_ < 0.5 < *y*_2_), using the equation *x* = [0.5–(*y*_1_ *x*_2_–*y*_2_ *x*_1_)/(*x*_2_–*x*_1_)]^*^(*x*_2_–*x*_1_)/(*y*_2_–*y*_1_).

### Quantitative real-time reverse transcription PCR (qRT-PCR)

HEK293 cells transfected with Na_v_1.7 (1 μ g) alone or with each of β-subunits (0.4 μ g) were collected in RNA-later solution (Qiagen, Basel, Switzerland). mRNA was extracted and purified with RNeasy Plus Mini kit (Qiagen) and quantified using RNA 6000 Nano Assay (Agilent Technologies AG, Basel, Switzerland). A total of 600 ng of RNA was reverse transcribed for each sample using Omniscript reverse transcriptase (Qiagen). Na_v_1.7 primer's sequence is as follow; 5′-TCTGTCTGAGTGTGTTTGCACTAA-3′ and 5′-AAGTCTTCTTCACTCTCTAGGGTATTC-3′. We used GAPDH as reference gene to normalize Na_v_1.7 mRNA expression. Gene-specific mRNA analyses were performed using the iQ SYBR-green Supermix (BioRad, Reinach, Switzerland) and the iQ5 real-time PCR detection system (BioRad). Only reactions with appropriate amplification and melting curves determining the amplicon specificity were analyzed. For all conditions tested we used *n* = 3 samples. All samples were run in triplicate.

### Statistics

For electrophysiological experiments (current densities and biophysical properties) normality with D'Agostino-Pearson was tested to determine whether a regular One-Way ANOVA and *post-hoc* Bonferroni tests, or the non-parametric equivalent test (Kruskal-Wallis test and Dunn *post-hoc* tests), should be performed. For RFI, a Two-Way ANOVA was used to compare Na_v_1.7 alone with Na_v_1.7 co-transfected with each β-subunit, and the impact of the voltage on this comparison. Biochemical experiments and transcriptional quantification data were analyzed using bilateral Student's *t*.

## Results

**Figure 1:**
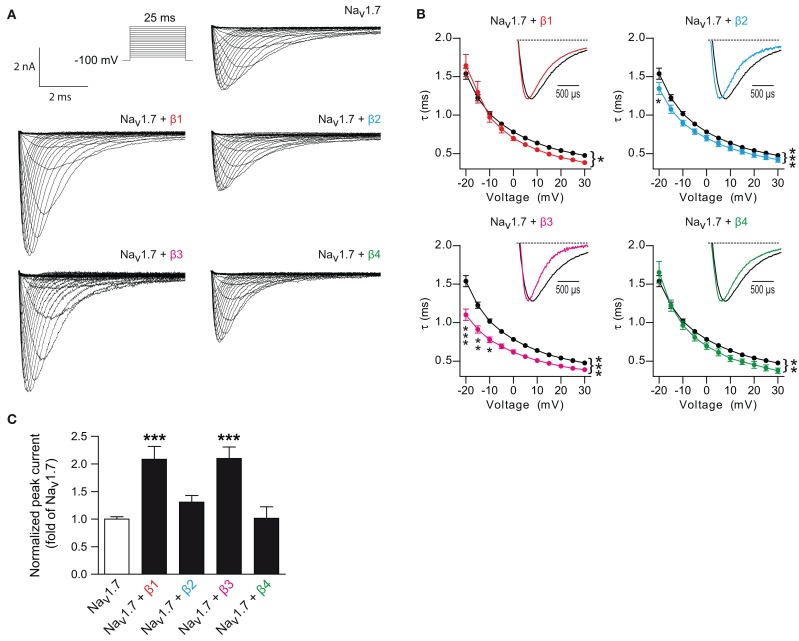
**β-subunits regulate Na_v_1.7 currents. (A)** Typical whole-cell Na^+^ currents of HEK293 cells transfected with Na_v_1.7 alone or Na_v_1.7 co-expressed with individual β-subunits elicited with a typical current-voltage protocol. **(B)** Voltage-dependence of current decay of Na_v_1.7 alone compared to Na_v_1.7 with each individual β-subunit. *Inset*: Normalized representative current traces of Na_v_1.7 elicited by test pulses at 0 mV. Co-transfection of the β1- (*p* = 0.011, *n* = 39), β2- (*p* < 0.0001, *n* = 25), β3- (*p* < 0.0001, *n* = 16), and β4-subunit (*p* = 0.006, *n* = 13) decreased the time constant decay as compared to Na_v_1.7 alone (*n* = 81). Two-Ways ANOVA and Bonferroni *post-hoc* tests. Data are expressed as mean ± s.e.m. **(C)** *I*_Na_ densities from HEK293 cells transfected with Na_v_1.7 alone or co-transfected with individual β-subunits. β1- (*n* = 55) and β3-subunits (*n* = 34), but not β2- (*n* = 67) nor β4-subunits (*n* = 27), increased the Na_v_1.7 current densities. *p* < 0.0001 for β1- and β3-subunits with One-Way ANOVA followed by Bonferroni *post-hoc* tests. Data are expressed as mean ± s.e.m. and were normalized to Na_v_1.7 alone for each experiments. Values can be found in Table 1. ^*^p < 0.05, ^**^p < 0.01 and ^***^p < 0.001.

The functional impact of the co-expression of the four β-subunits on Na_v_1.7-mediated *I*_Na_ was studied by performing whole cell patch-clamp experiments in HEK293 cells. Each β-subunit was independently co-transfected with Na_v_1.7 and then compared to Na_v_1.7 expressed alone. Figure 1A shows typical traces of Na_v_1.7 *I*_Na_ obtained with a current-voltage protocol. A hastening of the Na_v_1.7 current decay kinetics was observed with each of the β-subunits tested (Figure 1B). The shortening of the Na_v_1.7 time constant of current decay was observed for a wide range of voltages and showed voltage-dependency for every β-subunit (Figure 1B). The shortening was particularly prominent for the β3-subunit. In addition, β1- and β3-subunits also significantly increased (~2-fold) Na_v_1.7-mediated current density as compared to Na_v_1.7 alone or to Na_v_1.7 co-expressed with β2- or β4-subunits (Figure 1C and Table 1). We also observed that β2 and β4-subunits did not antagonize β1 and β3-subunits-dependent up-regulation, and that the two latter have additive positive effect on Na_v_1.7-mediated current (data not shown).

**Table 1:** **Biophysical properties of Na_v_1.7 alone or upon β-subunit co-transfection in HEK293 cells**.

	**Current density**		**Activation**			**Inactivation**			**Recovery**	
	**normalized pA/pF**	***n***	***V*_½_ (mV)**	**slope *k_v_***	***n***	***V*_½_ (mV)**	**slope *k_v_***	***n***	***t*_½_ (ms)**	***n***
Na_v_1.7	1.00 ± 0.04	140	−18.6 ± 0.4	6.8 ± 0.2	81	−70.9 ± 0.5	8.1 ± 0.3	92	7.55 ± 0.21	72
Na_v_1.7 + β1	2.08 ± 0.23^***^	55	−17.4 ± 0.8	5.8 ± 0.2^***^	39	−65.7 ± 0.5^***^	7.0 ± 0.2^*^	51	6.19 ± 0.23^**^	27
Na_v_1.7 + β2	1.31 ± 0.12	67	−18.2 ± 0.9	6.2 ± 0.3	25	−70.9 ± 0.6	7.5 ± 0.2	43	7.03 ± 0.34	34
Na_v_1.7 + β3	2.10 ± 0.21^***^	34	−22.3 ± 1.0^*^	5.4 ± 0.3^***^	17	−67.4 ± 0.8^***^	7.0 ± 0.3	18	6.69 ± 0.25	16
Na_v_1.7 + β4	1.02 ± 0.21	27	−16.1 ± 1.1	7.1 ± 0.3	13	−70.8 ± 0.5	7.2 ± 0.5	20	7.38 ± 0.33	19

Values for Na_v_1.7 and Na_v_1.7 co-transfected with β-subunits. The V_½_ of steady-state activation and inactivation and their associated slope factors, as well as the t_½_ of recovery from inactivation, were obtained as described in the Materials and Methods.

* p < 0.05,

** p < 0.01, and

*** p < 0.001, One-Way ANOVA, post-hoc Bonferroni tests or Kruskal–Wallis with Dunn post-hoc test between Na_v_1.7 and Na_v_1.7 with each β-subunit. Data are expressed as mean ± s.e.m.

**Figure 2:**
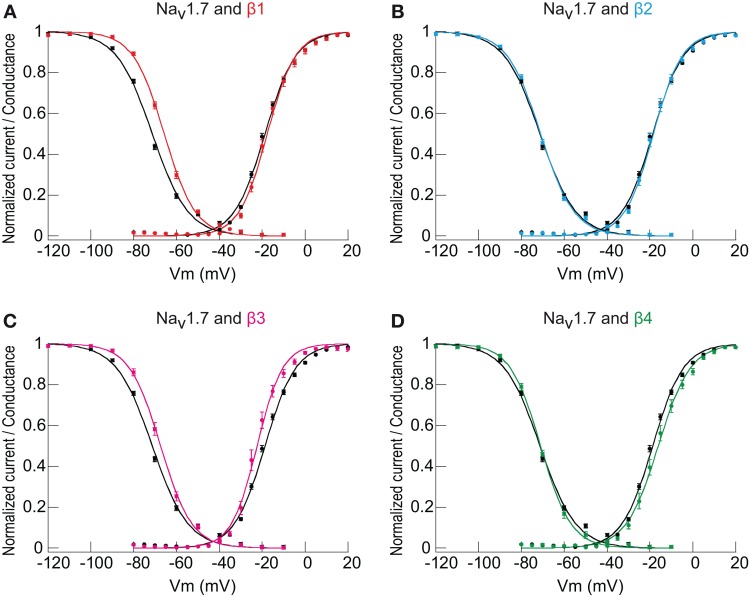
**The influence of β1- and β3-subunits on Na_v_1.7 voltage-dependence of activation and inactivation. (A–D)** Normalized currents for both activation and steady-state inactivation (see Materials and Methods) are plotted against the test potential. Each panel compares Na_v_1.7 alone to Na_v_1.7 co-expressed with individual β-subunits. A minor but significant effect on the V_½_of activation was observed for β3-subunit (*n* = 17, hyperpolarizing shift, *p* < 0.05) co-expression as compared to Na_v_1.7 alone (*n* = 81). β1- (*n* = 39), β2- (*n* = 25) and β4-subunits (*n* = 13) did not modify the *V*_½_of activation of Na_v_1.7. The effect on the *V*_½_ of inactivation was highly significant for β1- (*n* = 51, depolarizing shift with *p* < 0.0001) and β3-subunit (*n* = 18, depolarizing shift with *p* < 0.0001) compared to Na_v_1.7 alone (*n* = 92); whereas β2- (*n* = 43) and β4-subunits (*n* = 20) had no effect. Individual points are the mean ± s.e.m. of the normalized current at each voltage point. The smooth curves are Boltzmann fits whose equations give both the *V*_½_of activation and inactivation (midpoints) and their associated slope factors (see Materials and Methods). The *V*_½_ of steady-state activation and inactivation comparing Na_v_1.7 alone vs. the co-expression with each subunit were tested by One-Way ANOVA followed by Bonferroni's multiple comparison tests. Values and statistics can be found in Table 1.

Whether the *I*_Na_ density increase mediated by both β1- and β3-subunits was also accompanied by alterations of other Na_v_1.7 biophysical properties was also assessed. The voltage dependence of macroscopic *I*_Na_ activation and inactivation (see Materials and Methods) of Na_v_1.7 in the absence and presence of each β-subunit was recorded and analyzed. The co-transfection of the β1-subunit significantly shifted the *V*_½_ of steady-state inactivation toward depolarized potentials by ~5.8 mV, but had no influence on *V*_½_ of activation (Figure 2A and Table 1). The β3-subunit shifted the *V*_½_ of inactivation toward depolarized potentials by ~3.5 mV and the *V*_½_ of activation toward hyperpolarized potentials by ~3.7 mV (Figure 2C and Table 1). Neither the β2- nor β4-subunits affected Na_v_1.7 voltage dependence of activation or inactivation (Figures 2B,D and Table 1).

**Figure 3:**
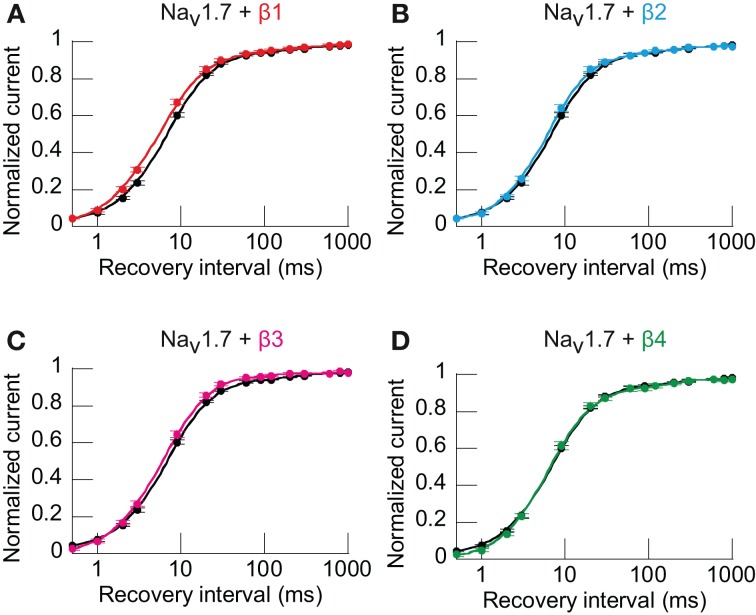
**Only β1-subunit significantly increases Na_v_1.7 recovery from inactivation (RFI). (A–D)** RFI from HEK293 cells transfected with Na_v_1.7 alone or co-transfected with individual β-subunits. Smooth curves were fitted incorporating a geometric weight to arrive at the final curve (no equation) and the *t*_½_ was calculated by interpolation on the *x*-axis from a linear relation between the 2 points juxtaposing half recovery (*y*_1_ < 0.5 < *y*_2_, see Materials and Methods). Only when co-expressed with β1-subunit (*n* = 27, *p* < 0.01) was Na_v_1.7 RFI significantly faster as compared to Na_v_1.7 alone (*n* = 72). β2- (*n* = 34), β3- (*n* = 16), and β4-subunits (*n* = 19) did not significantly alter RFI when co-expressed with Na_v_1.7. Individual points are the mean ± s.e.m. of the normalized current at each time point. Non-parametric One-Way analysis of variance (Kruskal-Wallis test) with Dunn *post-hoc* tests to compare each subunit co-expressed with Na_v_1.7 vs. Na_v_1.7 alone.

The influence of the β-subunits on recovery from inactivation (RFI) was also tested. Because the RFI relationships could not always be fitted with the exponential functions to the same degree, an interpolation from a linear relation between the 2 points juxtaposing half recovery to obtain the half-time (*t*_½_) of RFI was used. Only the β1-subunit significantly hastened *t*_½_ of RFI to 6.19 vs. 7.55 ms for the control (Figures 3A–D and Table 1).

**Figure 4:**
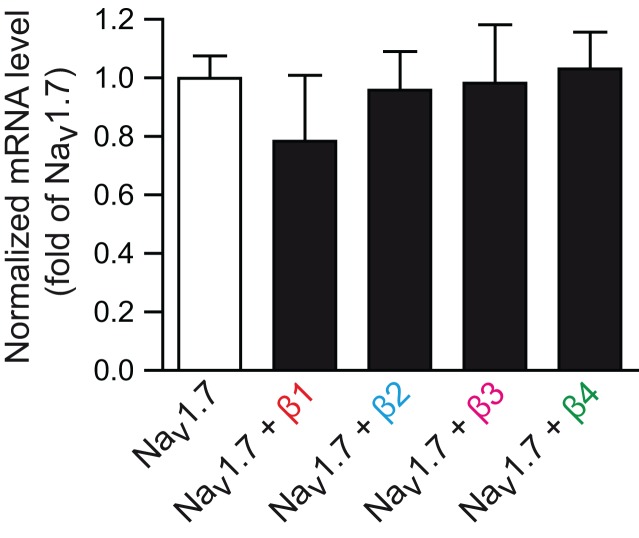
**Na_v_1.7 transcripts upon β-subunit co-transfection**. Bar graph showing transcriptional levels of Na_v_1.7 in control conditions (co-transfected with empty vector) over Na_v_1.7 levels when cells were co-transfected with each individual β-subunit. Transcripts were normalized to *GAPDH* and run in triplicate. Data represent the mean ± s.e.m., *n* = 3 independent transfections for every condition.

As only minor modifications of the *I*_Na_ biophysical properties were observed, it is unlikely that the 2-fold increase in the Na_v_1.7 current density mediated by β1- and β3-subunits is only due to alterations of the single channel properties. Whether the increase of the Na_v_1.7 current may have been due to an increase in channel synthesis was investigated. Na_v_1.7 mRNA levels remained unchanged with β-subunit co-transfection, as observed by q-RT-PCR (Figure 4), discounting this hypothesis.

**Figure 5:**
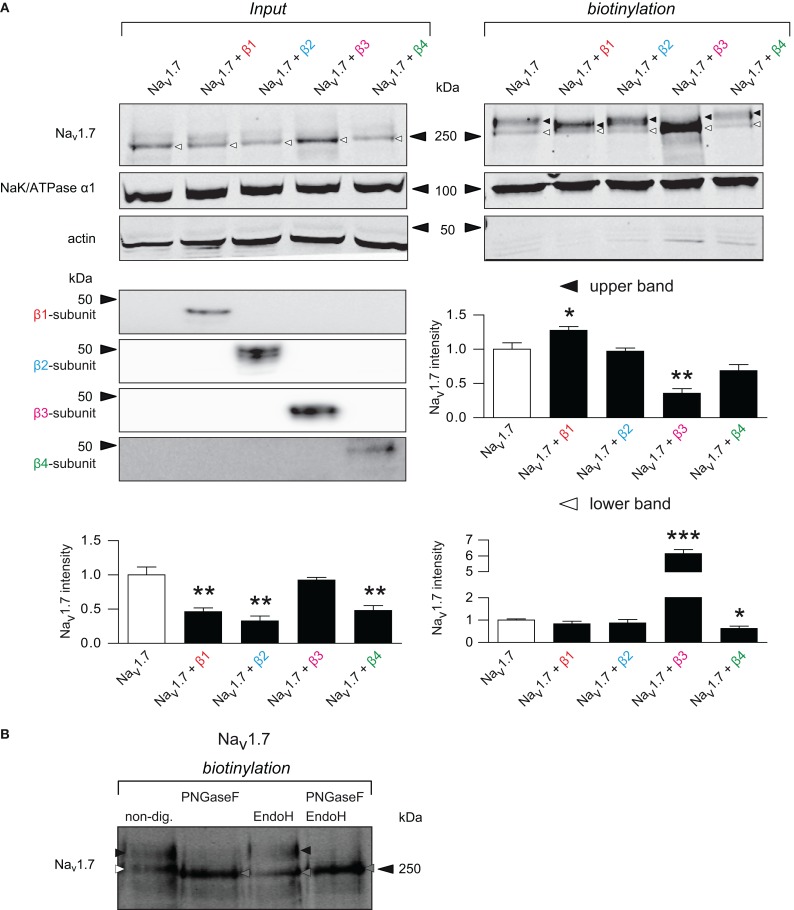
**β1- and β3-subunit mediate differential forms of Na_v_1.7 whose expression is increased at the membrane. (A)** Representative western blot of a biotinylation assay with total lysate (*input*, left) and cell surface (*biotinylation*, right) fractions from HEK293 cells transiently transfected with Na_v_1.7 alone, or co-expressed with each individual β-subunit and the associated quantifications. *Input:* Na_v_1.7 is detected in two forms: a fast migrating band (~250 kDa, that will be referred to as lower band) that consist mostly of the Na_v_1.7 immunoreactive signal and a slow migrating band (~280 kDa, that will be referred to as upper band). β1- (*p* = 0.006), β2- (*p* = 0.003), and β4-subunits (*p* = 0.009) significantly decreased Na_v_1.7 expression, whereas the β3-subunit had no effect (*p* = 0.570). Because the upper band was below the sensitivity threshold, both bands were quantified together. *Biotinylation:* Na_v_1.7 membrane protein is detected in three forms. When expressed alone, one lower band (white triangle, ~250 kDa) and one upper band (black triangle, ~280 kDa) were present (for identification of these bands, see Panel **B**). When the β1-subunit is co-expressed, the upper band was clearly shifted into an intermediate migrating band (~260 kDa) with increased expression (*p* = 0.047). β2- and β4-subunits revealed the same pattern as when Na_v_1.7 was transfected alone and did not change its expression, except for the small decrease of the lower band when the β4-subunit is co-transfected (*p* = 0.020). The β3-subunit clearly increased Na_v_1.7 immunoreactivity of the lower band (*p* < 0.0001). For *input* and *biotinylation* fractions, actin and the α1-subunit of NaK-ATPase were used as biotin leakiness and loading controls, respectively. Data represent mean ± s.e.m, *n* = 4 independent experiments. Student's unpaired *t*-test, each condition being compared with Na_v_1.7. ^*^p < 0.05, ^**^p < 0.01 and ^***^p < 0.001. **(B)** Representative western blot and identification of glycosylation state of Na_v_1.7 in biotinylated fraction from HEK293 cells transiently transfected with Na_v_1.7. EndoH only cleaves the lower band of biotinylated Na_v_1.7, demonstrating that this band represents the core-glycosylated form of the channel. The upper band is digested by PNGaseF, demonstrating that it corresponds to fully-glycosylated form of the channel. PNGaseF can also digest the core-glycosylated form of Na_v_1.7.

Whether the Na_v_1.7-mediated *I*_Na_ upregulation could be due to an increase of Na_v_1.7 protein density at the cell membrane was investigated by performing biotinylation of plasma membrane proteins. After lysis, proteins were sampled under reducing conditions known to dissociate the covalently bound β2- and β4-subunits from α-subunits (Messner and Catterall, 1985). Co-transfection of β1-, β2-, and β4-subunits significantly decreased Na_v_1.7 protein expression in the total cell lysate fraction (input, Figure 5A). The quantification revealed a ~2-fold decrease for each of these three subunits. Co-transfection of the β3-subunit had no effect on Na_v_1.7 expression in the total cell lysate fraction. In the biotinylated membrane fraction two bands at different apparent molecular weights were observed when Na_v_1.7 was expressed alone (white and black arrow heads in Figure 5A). These bands correspond to different glycosylated states of Na_v_1.7 as demonstrated by using deglycosylating enzymes (Figure 5B). Endoglycosidase H (EndoH) only cleaves core N-glycans from proteins whereas Peptide-N-Glycosidase F (PNGaseF) does not discriminate between full and core glycosylated proteins. Of the two bands of biotinylated Na_v_1.7, only the lower was sensitive to EndoH and was shifted to an apparent lower molecular weight band (compare white arrowhead in the first lane to gray arrowhead in the third lane), indicating that this band corresponds to the core-glycosylated form of the channel (Figure 5B). Because PNGaseF was able to digest both bands, it can be proposed that the higher band corresponds to the fully-glycosylated form of Na_v_1.7. The lower band of biotinylated Na_v_1.7 migrates at the same apparent molecular weight as the band observed in the input fraction (white arrow heads in Figure 5A) suggesting that most of Na_v_1.7 in the intracellular pool is core-glycosylated. This is consistent with the channel being early and rapidly, but only partially, glycosylated after its synthesis. The upper band in the total cell lysate fraction was faint and blurry (Figure 5A), suggesting that the fully-glycosylated channel only represents a small fraction of the total Na_v_1.7 cellular pool. It was only by enriching the membrane proteins through the precipitation of the biotinylated membrane fraction (the ratio between the amount of lysate protein loaded and the amount of streptavidin beads needed to precipitate biotinylated proteins was ~1:30) that the upper band was distinctly observed. Co-expression of the β1-subunit reproducibly shifted the upper band to an intermediate migrating band of lower apparent molecular weight. This suggests that the β1-subunit mediates an alternative glycosylated form of Na_v_1.7. When comparing the β1-subunit-modified intermediate band with the upper band of the control condition (Na_v_1.7 alone), a significant increase in signal intensity was observed (Figure 5A, quantification), which is consistent with the increase in the Na_v_1.7 current density (Figure 1C). Co-transfection of the β2-subunit neither modified the glycosylation pattern nor the expression of any of the two bands, consistent with the fact that the current density was not modified. β3-subunit expression also altered the Na_v_1.7 band pattern in the biotinylated fractions. The upper band overlapped with the lower band under the migrating conditions used. The β3-subunit significantly increased (~7-fold) the intensity of the lower band as compared to the lower band of control, consistent with the increase of the Na_v_1.7 current density elicited by the β3-subunit (Figure 1C). Finally, β4-subunit co-transfection led to a small but significant decrease of the lower band.

**Figure 6:**
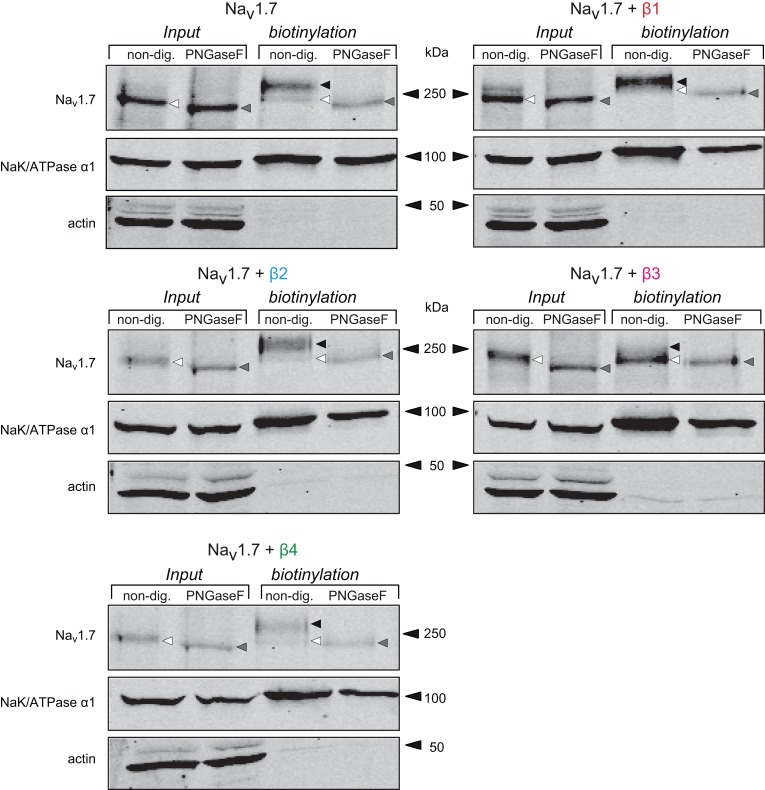
**The different forms of Na_v_1.7 observed with β-subunits are due to differential glycosylation patterns**. Western blot of a biotinylation assay followed by deglycosylation with total lysate and cell surface fractions from HEK293 cells transiently transfected with Na_v_1.7 alone, or co-expressed with each individual β-subunit. Samples were non-treated or treated with Peptide: N-Glycosidase F (PNGaseF) to remove glycosylated residues of the protein. The total lysate Na_v_1.7 band (black/white triangle) was slightly shifted to an apparent lower molecular weight (gray triangle) when treated with PNGaseF. In the biotinylation fraction, the pattern of Na_v_1.7 glycosylation by the β-subunits was the same as in Figure 4.(black and white triangles). When treated with PNGaseF, all the different bands shifted to the lower band of the same apparent molecular weight, irrespective of the β-subunit co-expressed.

To confirm that the different bands observed when β-subunits are coexpressed, particularly β1 and β3-subunits, represent alternative glycosylated form of Na_v_1.7, we again incubated the input and biotinylated fractions with PNGaseF. A small but consistent shift of the Na_v_1.7 band into a lower apparent molecular weight band in the total cell lysate fraction was observed (the white arrow heads shifted to the gray arrow heads, Figure 6). Furthermore, when incubating the biotinylated fraction of β-subunit and Na_v_1.7 co-expression experiments with PNGaseF, all of the Na_v_1.7 bands shifted to a single band (gray arrow heads) of the same molecular weight. This confirms that the β1- and β3-subunits modulate differential glycosylation patterns on Na_v_1.7 (Figure 6, black and white arrow heads). Noteworthy, when comparing the single band of Na_v_1.7 when samples are treated with PNGaseF in the input fraction with the one in the biotinylated fraction, it seems that this band migrates slower in the input as compared to biotinylated fraction when β-subunits are coexpressed (compare bands highlighted by gray arrows for each blots). This may be due to other post-translational modification such as sialylation or palmitoylation of the channel. Further experiments using desialylation or depalmitoylation treatment are needed to confirm this possibility.

## Discussion

This study demonstrates that Na_v_ β1- and β3-subunits modulate the cell surface expression and glycosylation patterns of Na_v_1.7 when co-expressed in HEK293 cells. It also confirms that the β-subunits differentially modulate the biophysical properties of Na_v_1.7.

The observation that β1- and β3-subunits strongly increased Na_v_1.7 *I*_Na_ density contrasts with several recent studies. The study performed by Ho et al. (2012) showed no impact of any of the β-subunits on Na_v_1.7 current density in HEK293 cells. The other studies showed no impact using different cell expression systems (Sangameswaran et al., 1997; Morgan et al., 2000; Vijayaragavan et al., 2001, 2004). These discrepant observations underline the influence of the cellular background when studying Na_v_s α-subunit regulation by β-subunits. Even though this study and the one by Ho et al. (2012) both used HEK293 cells, Ho et al., used a clonal cell line stably expressing rat Na_v_1.7 cDNA, whereas the present study used transiently transfected human Na_v_1.7 cDNA. In a stable cell line, it is likely that a significant fraction of α-subunits are already anchored at the plasma membrane; thus it is possible that the ones interacting with the transfected β-subunits only reflect a small fraction of membrane Na_v_s α-subunits. Under the conditions of transient transfection, all the membrane Na_v_s α-subunits are synthesised *de novo* and are thus more likely to interact with β-subunits. It is also possible that other differences, such as the origin of the HEK293 cells or even passage numbers, may play a significant role in the observed effects. Other studies have shown, however, that β1- and β3-subunits can increase the current density of several other Na_v_ isoforms (Nuss et al., 1995; Smith and Goldin, 1998; Fahmi et al., 2001; Zimmer and Benndorf, 2002).

A significant shortening of the time constant of current decay was observed when Na_v_1.7 was co-expressed with each individual subunit. A rapid rate of inactivation tends to reduce the refractory period of Na_v_1.7, meaning that β-subunits can enhance cell excitability via this mechanism. The hastening of RFI by the β1-subunit might also reduce the duration of the refractory periods, allowing for faster repetitive firing of neurons.

The β1-subunit shifted *V*_½_ of inactivation toward more depolarized potentials, which should increase the number of channels available for opening in response to depolarization at a given voltage near the resting membrane potential (approximately −60 to −70 mV). The β3-subunit similarly influenced this parameter, and also shifted the *V*_½_ of activation toward hyperpolarized potentials, rendering the channel more likely to open at hyperpolarized voltages. These results are consistent with the findings of Ho et al., and account for the shift toward a hyperexcitable state.

The fact that the β1- and β3-subunits strongly increased Na_v_1.7 current density, but only modestly influenced the biophysical properties, suggests that the single-channel conductance is not altered. In line with the previous, single-channel recordings revealed that β1-subunit did not change the Na_v_1.5 open probability despite an important increase in current density (Nuss et al., 1995). As a consequence, it was hypothesized that these two subunits also increase Na_v_1.7 channel density at the cell surface. Biotinylation of cell surface proteins was performed and a strong decrease of the Na_v_1.7 signal in the input fraction when β1-, β2-, and β4-subunits were co-transfected was observed. This decrease was in contrast with the increase or lack of modification of the Na_v_1.7 current density. The reason for this decrease remains to be identified, but one can speculate that the expression of the β-subunits may decrease the ratio of intracellular Na_v_s to plasma membrane Na_v_s by hastening forward trafficking and stabilizing the channel at the cell surface. Furthermore, one of the important functions of glycosylation is the proper folding and protection of proteins with respect to proteolysis (Parodi, 2000). The thus far not observed role of β-subunits in altering Na_v_1.7 glycosylation might influence the degradation by the proteasome, accounting for the decrease in input. The β-subunits have also been proposed to act as chaperon proteins (Valdivia et al., 2010), further supporting a potential effect on degradation by the proteasome.

The analysis of the Na_v_1.7 protein at the plasma membrane and the subsequent treatment with the deglycosylating enzyme led to three novel findings: (1) Under normal conditions, Na_v_1.7 is present in two glycosylated forms, a core-glycosylated form with a molecular weight of ~250 kDa and a fully-glycosylated form with a molecular weight of ~280 kDa; (2) The β1-subunit can further mediate a third and intermediate migrating band, which likely represents an alternative fully-glycosylated form of Na_v_1.7; and (3) the β1-subunit increases the membrane expression of this alternative fully-glycosylated form of Na_v_1.7; whereas the β3-subunit increases the membrane expression of the core-glycosylated form.

In studies using chimeras between the β1- and β3-subunits, it was proposed that different parts of these subunits were involved in the modulation of gating by direct interaction with the α-subunits (Zimmer and Benndorf, 2002). The observation in this study that the β1- and β3-subunits can mediate the differential glycosylation of Na_v_1.7, and despite the well-documented causal link demonstrating that differential glycosylation leads to a modification of Na_v_s α-subunit gating (Bennett et al., 1997; Zhang et al., 1999; Tyrrell et al., 2001), does not allow us to conclude that the β-subunit-mediated glycosylation of the α-subunit is responsible for the modulation of Na_v_1.7 gating. Further studies to identify the potential glycosylation site of Na_v_1.7 are necessary to determine whether the effects of the β-subunits on the biophysical properties of Na_v_1.7 are also dependent on this mechanism. Nevertheless, the observation that the β1- and β3-subunits influence the gating properties of Na_v_1.7 and alter its glycosylation pattern, while the β2- and β4-subunits do not, supports this hypothesis.

It was recently proposed that only the upper band of biotinylated Na_v_1.7 may reflect the functional fully-glycosylated form of the channel, whereas the lower band represents an intermediate and/or immature glycosylated form of the channel which does not participate in Na^+^ conductance (Laedermann et al., 2013). The upregulation of expression of the core-glycosylated form of Na_v_1.7 when the β3-subunit is co-transfected, along with the associated 2-fold increase in the Na_v_1.7 current density, suggests that there is no such dichotomy and that the link between the glycosylation and functionality of the channel is more complex. It is possible that these distinctly glycosylated forms of Na_v_1.7 differentially participate in Na^+^ conductance, but their relative contribution to the overall sodium current has yet to be determined. For instance, quantification of the shifted upper band when the β1-subunit was present revealed an increased signal intensity of ~30%, which is less important than the 100% increase of *I*_Na_ measured using the patch clamp approach. This underlines that, in addition to an increased stabilization of the channel, modification of single channel conductance by the intermediate glycosylated form of Na_v_1.7 may also partially contribute to the functional 2-fold increase in *I*_Na_. This point, in addition to the fact that β-subunits have the ability to shift bands from one glycosylated state to another, demonstrates that quantification of biotinylated proteins needs to be interpreted with caution. It cannot be excluded that due to these shifts, some bands might contaminate the signal of another band.

**Figure 7:**
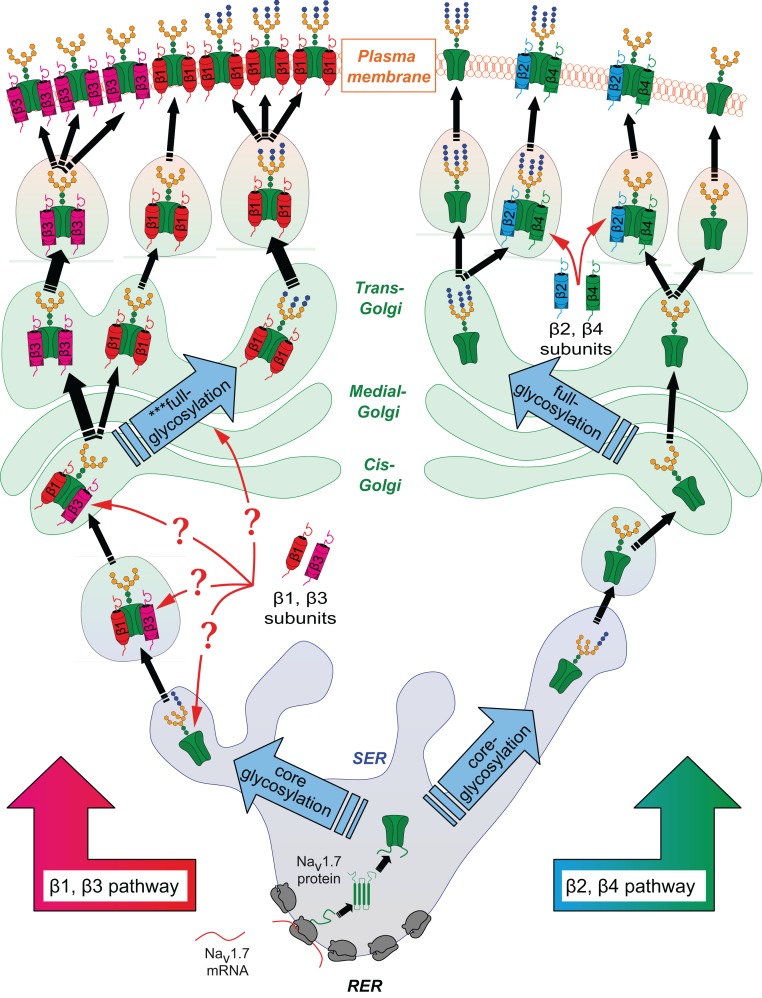
**Proposed scheme of the different intracellular pathways of α-subunits depending on the presence of different β-subunits**. After synthesis in the rough endoplasmic reticulum (RER), α-subunits are rapidly folded and undergo a first step of glycosylation in the smooth endoplasmic reticulum (SER). It is known that N-acetlyglucosamine and oligosaccharide chains are bound on Asp residues of the protein, a process known as core-glycosylation. Newly synthesized glycoproteins are then translocated into the Golgi network, where they are subject to a second step of more complex glycosylation, involving many different enzymes. Once matured, proteins eventually translocate to the plasma membrane. The present findings suggest that core-glycosylated proteins can also be found anchored at the membrane. By some yet undefined mechanism, the β1-subunit interferes with the second glycosylation step (^***^full-glycosylation on the scheme) which leads to a modification of the glycosylation pattern of the α-subunits. The β1-subunit enhances the core-glycosylated form of Na_v_1.7. This suggests that the β1- and β3-subunits already interact with the α-subunits before the step of full-glycosylation of the channel occurring in the Golgi network. By enhancing the differential glycosylation pattern of Na_v_1.7, it can be proposed that the β1- and β3-subunits promote stabilization of the channel at the plasma membrane. On the contrary, it is likely that the β2- and β4-subunits, which have no effect on the glycosylation nor the anchoring, only briefly interact with the α-subunits before translocation of the channel to the plasma membrane. For sake of simplicity, the shown glycosylation patterns are arbitrary. Na_v_1.7 is depicted as being “freely” expressed in ER/Golgi and membrane networks for easier interpretation of the scheme. However, Na_v_1.7 is embedded in the membranes of the different organelles.

General kinetic models of biosynthesis (Schmidt and Catterall, 1987) have proposed that the β2-subunit interacts with the α-subunit right before anchoring at the membrane. The present findings are consistent with such a model since both β2- and β4-subunits did not influence Na_v_1.7 glycosylation, and most likely interacted after the Golgi network (Figure 7). The α−β1 complex was previously shown to associate in the ER, enhancing the trafficking to the plasma membrane (Zimmer et al., 2002). Another study demonstrated that β1- and β3-subunits increase the efficiency of channel trafficking from the ER to the plasma membrane (Fahmi et al., 2001). The β3-subunit has also been shown to mask the ER-retention signal (Zhang et al., 2008). The results of the present work confirm earlier demonstrated interactions between the β- and α-subunits, and suggest an additional function of both: β1- and β3-subunits interact with the α-subunit in the ER/Golgi, where they regulate the differential glycosylation of Na_v_ α-subunits, which in turn modulates the stabilization of the channel at the cell membrane (Figure 7). The mechanisms underlying their interaction and mediation of α-subunit glycosylation, as well as the identification of other potential isoforms that would be subject to such regulation, remain to be investigated.

### Physiological relevance

These results were obtained in cellular expression system. Whether such mechanisms also occur in native cells remain to be investigated. This study used the Na_v_1.7 isoform, an important contributor to pain processing (Lampert et al., 2010). The electrophysiological results show that β1- and β3-subunits are able to increase Na_v_1.7 excitability, as demonstrated by the increase in peak current, kinetics, voltage-availability and repriming rate. Na_v_1.7 is expressed in high levels in all types of sensory neurons (Ho and O'leary, 2011). β-subunits are also expressed in sensory neurons, but to a variable extent depending on cell type (Takahashi et al., 2003; Ho et al., 2012). Furthermore, β1- and β3-subunit expression has been reported to be increased in pathological pain (Shah et al., 2000; Coward et al., 2001), where they have been implicated in the generation of hyperexcitability. It remains to be determined if Na_v_ function is mediated by an altered pattern of α-subunit glycosylation, which is an important regulatory process of excitability in dorsal root ganglia neurons (Tyrrell et al., 2001).

### Conflict of interest statement

The authors declare that the research was conducted in the absence of any commercial or financial relationships that could be construed as a potential conflict of interest.
