# Supplementary material for: β1- and β3- voltage-gated sodium channel subunits modulate cell surface expression and glycosylation of Nav1.7 in HEK293 cells
Source: Front Cell Neurosci. 2013 Aug 30;7:137. doi: 10.3389/fncel.2013.00137 (PMC3757325; doi:10.3389/fncel.2013.00137)
Supplement: Supplementary file 1 [file Presentation1.PPT]

## Slide 1
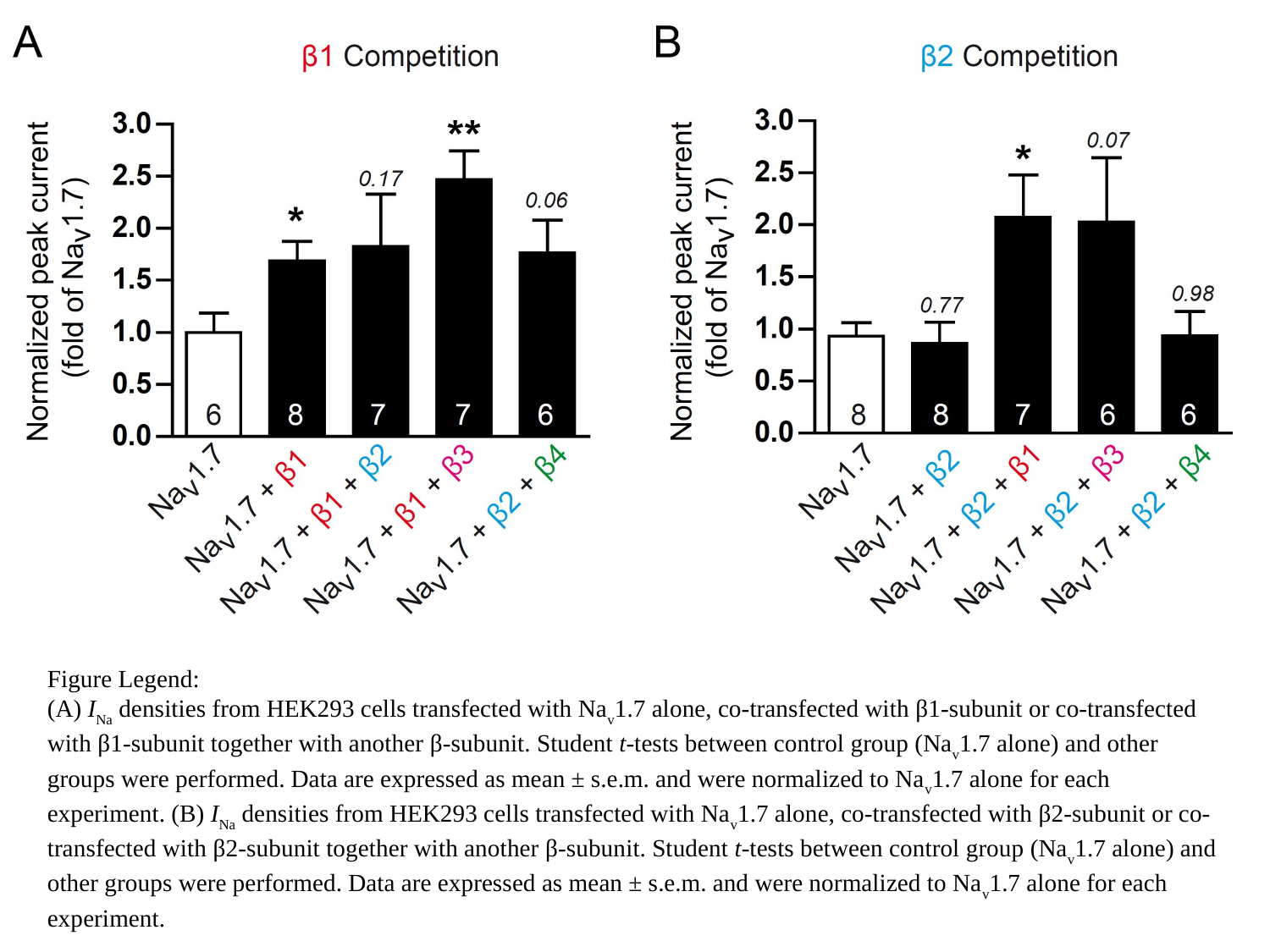

Figure Legend:
(A) INa densities from HEK293 cells transfected with Nav1.7 alone, co-transfected with β1-subunit or co-transfected with β1-subunit together with another β-subunit. Student t-tests between control group (Nav1.7 alone) and other groups were performed. Data are expressed as mean ± s.e.m. and were normalized to Nav1.7 alone for each experiment. (B) INa densities from HEK293 cells transfected with Nav1.7 alone, co-transfected with β2-subunit or co-transfected with β2-subunit together with another β-subunit. Student t-tests between control group (Nav1.7 alone) and other groups were performed. Data are expressed as mean ± s.e.m. and were normalized to Nav1.7 alone for each experiment.
